# Machine Learning in Cardio-Oncology: New Insights from an Emerging Discipline

**DOI:** 10.31083/j.rcm2410296

**Published:** 2023-10-19

**Authors:** Yi Zheng, Ziliang Chen, Shan Huang, Nan Zhang, Yueying Wang, Shenda Hong, Jeffrey Shi Kai Chan, Kang-Yin Chen, Yunlong Xia, Yuhui Zhang, Gregory Y.H. Lip, Juan Qin, Gary Tse, Tong Liu

**Affiliations:** ^1^Tianjin Key Laboratory of Ionic-Molecular Function of Cardiovascular Disease, Department of Cardiology, Tianjin Institute of Cardiology, Second Hospital of Tianjin Medical University, 300211 Tianjin, China; ^2^National Institute of Health Data Science at Peking University, Peking University, 100871 Beijing, China; ^3^Institute of Medical Technology, Peking University Health Science Center, 100871 Beijing, China; ^4^Cardio-Oncology Research Unit, Cardiovascular Analytics Group, PowerHealth Limited, 999077 Hong Kong, China; ^5^Department of Cardiology, First Affiliated Hospital of Dalian Medical University, 116011 Dalian, Liaoning, China; ^6^Heart Failure Center, State Key Laboratory of Cardiovascular Disease, Fuwai Hospital, National Center for Cardiovascular Diseases, Chinese Academy of Medical Sciences and Peking Union Medical College, 100037 Beijing, China; ^7^Liverpool Centre for Cardiovascular Science, University of Liverpool, Liverpool John Moores University and Liverpool Heart & Chest Hospital, L69 3BX Liverpool, UK; ^8^Danish Center for Health Services Research, Department of Clinical Medicine, Aalborg University, 999017 Aalborg, Denmark; ^9^Section of Cardio-Oncology & Immunology, Division of Cardiology and the Cardiovascular Research Institute, University of California San Francisco, San Francisco, CA 94143, USA; ^10^School of Nursing and Health Studies, Hong Kong Metropolitan University, 999077 Hong Kong, China

**Keywords:** cardio-oncology, machine learning, cardiotoxicity, inequity, multidisciplinary team

## Abstract

A growing body of evidence on a wide spectrum of adverse cardiac events following oncologic therapies has led to the emergence of cardio-oncology as an increasingly relevant interdisciplinary specialty. This also calls for better risk-stratification for patients undergoing cancer treatment. Machine learning (ML), a popular branch discipline of artificial intelligence that tackles complex big data problems by identifying interaction patterns among variables, has seen increasing usage in cardio-oncology studies for risk stratification. The objective of this comprehensive review is to outline the application of ML approaches in cardio-oncology, including deep learning, artificial neural networks, random forest and summarize the cardiotoxicity identified by ML. The current literature shows that ML has been applied for the prediction, diagnosis and treatment of cardiotoxicity in cancer patients. In addition, role of ML in gender and racial disparities for cardiac outcomes and potential future directions of cardio-oncology are discussed. It is essential to establish dedicated multidisciplinary teams in the hospital and educate medical professionals to become familiar and proficient in ML in the future.

## 1. Introduction

In recent years, advances in cancer diagnosis and treatment have significantly improved the survival and quality of cancer patients. However, this has been accompanied by a significant increase in the incidence of cardiotoxicity associated with cancer therapies [1, 2, 3]. A population-based study conducted on the causes of cardiovascular disease (CVD) death in the US has found that among 3.2 million cancer survivors, 38.0% eventually died from cancer and 11.3% died from CVDs, with 76.3% of CVD deaths were caused by heart disease [4]. CVD has become a significant cause of mortality and morbidity among cancer survivors [4]. CVD and malignancy share common risk factors, including age, obesity, and diabetes mellitus, and biological mechanisms such as increased oxidative stress and a pro-inflammatory milieu [5, 6]. Their clinical convergence led to the emerging discipline of cardio-oncology, which mainly focuses on the detection, monitoring, and treatment of cardiovascular disease occurring in the context of cancer treatment, encompassing both chemotherapy and radiotherapy.

Over the past decade, artificial intelligence (AI), particularly machine learning (ML), has changed medical practice and research to some degree [7]. By leveraging massive amounts of data, AI provides personalized opportunities for disease diagnosis, classification, risk stratification, and management [8]. Unlike human-coded time-to-event analysis, which relies on the expertise of the researcher to develop accurate and reliable coding criteria, ML algorithms use complex mathematical models to automatically identify patterns in the data. Statistical methods used in ML include regression analysis, clustering, and classification. Regression analysis enables the modeling of the relationship between a dependent variable and one or more independent variables. Clustering, on the other hand, is utilized to group similar data points into clusters, whereas classification is a technique for categorizing data points into distinct classes based on their features or characteristics [9]. ML algorithms often require large datasets to train and test the models. This is because the accuracy and effectiveness of ML models often increase with the amount of data used by them. Using data-trained learning algorithms, ML can make judgments about new situations, including but not limited to evaluating radiographic images, electronic medical records and pathology slides [10].

There are many branches of ML, including random forest (RF), artificial neural networks (ANN), convolutional neural networks and deep learning (DL) [11, 12], each having unique properties useful in cardio-oncology. ANN can simulate human neurons and process electrocardiogram (ECG) and echocardiographic data. In the cardiovascular field, the application of ANN mainly focuses on the multi-layer ANN used to simulate the human brain to operate in DL. It is also widely used in the analysis of imaging data, drug dosing and patient survival [12, 13, 14, 15]. Moreover, RF is commonly used in coronary computed tomography (CT) image processing, readmission of patients with heart failure, and the development of prediction models [16, 17, 18].

**Fig. 1.**
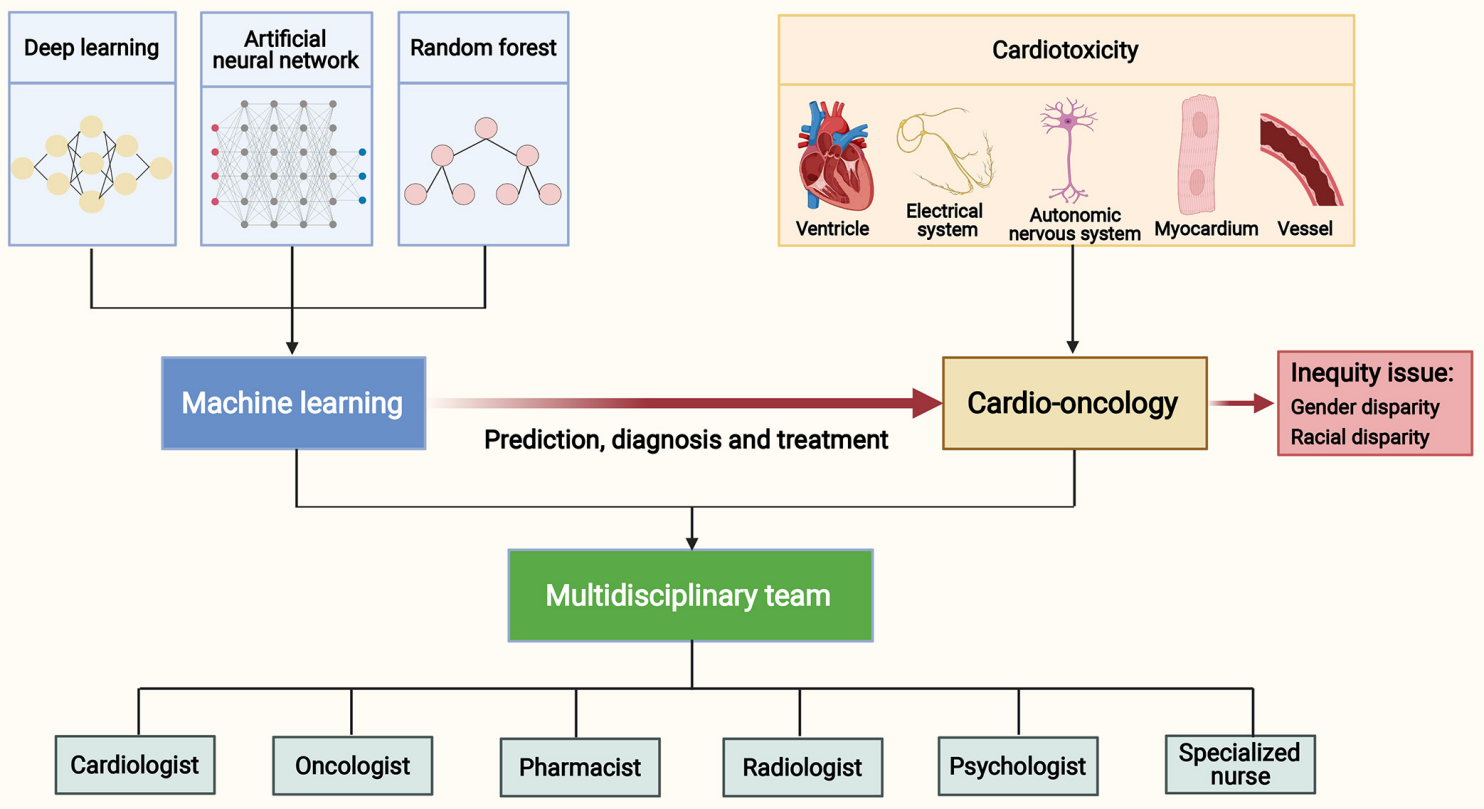
**Machine learning in cardio-oncology and a multidisciplinary team.** Anticancer therapies may cause cardiotoxicities to the ventricle, electrical system, autonomic nervous system, myocardium and vessel. Machine learning approaches, including deep learning, artificial neural network and random forest, have an exciting application in the prediction, diagnosis and treatment of cardiotoxicities. Comprehensive management of cancer patients with cardiovascular diseases requires an equitable approach and the development of multidisciplinary teams. These teams should include experts such as cardiologists, oncologists, pharmacists, radiologists, psychologists, and specialized nurses.

In this article, we systematically review the application of ML approaches in cardio-oncology, comprehensively describe the cardiotoxicities identified by ML, and outline the role of ML in the prediction, diagnosis and treatment of cardiotoxicities in cancer patients. In addition, we discuss the role of ML in better understanding the gender and racial disparities for cardiac outcomes among cancer patients. Given the current application of ML in clinical practice, we provide a perspective on the future development direction and challenges in cardio-oncology (Fig. 1).

## 2. ML Approaches in Cardio-Oncology

Iatrogenic treatment harm refers to the harm caused by medical treatments or procedures [19]. In the context of cancer treatment, iatrogenic harm refers specifically to the harm caused by cancer treatments, such as chemotherapy, radiation therapy, and surgery. These treatments can result in both direct and indirect damage to the heart and cardiovascular system, and leading to a range of complications [20]. ML has the potential to significantly reduce the iatrogenic treatment harm. By analyzing large and complex datasets of patient information, ML algorithms can detect patterns and correlations that may not be easily discernible by humans [21]. This analytical capability can help predict which patients are at a higher risk of experiencing cardiotoxicity from cancer treatments, thus enabling healthcare providers to implement targeted interventions and personalized care. Different ML approaches in cardio-oncology are discussed as follows.

### 2.1 Deep Learning

DL is a type of ML that combines statistics, computer science, and decision theory [22]. It is useful for analyzing hemodynamic and electrophysiological metrics which are increasingly obtained through wearable devices and cardiac imaging segmentation [22, 23, 24, 25, 26]. DL has shown exciting potential in automating complex image analysis [27], with broad applications in ultrasound, computed tomography, and magnetic resonance imaging [28]. The application of ML approaches in cardio-oncology is summarized in Table 1 (Ref. [29, 30, 31, 32, 33, 34, 35, 36, 37, 38, 39, 40]).

**Table 1.** **ML approaches in cardio-oncology**.

ML types	Sample	Cancer types	Conclusions	Reference
DL	273	Lung cancer	∙The ML-left atrial volume index was significantly associated with a higher risk of new-onset atrial fibrillation, heart failure hospitalization, and major adverse cardiovascular events within five years in patients who underwent LDCT.	[29]
DL	2085	Lung cancer	∙Application of DL to LDCT could be a dual assessment tool for the risk of lung cancer and CVDs.	[30]
DL	180	Lung cancer	∙It was feasible to use DL techniques to predict all-cause mortality from chest LDCT images in lung cancer patients.	[31]
DL	55	NSCLC	∙DL can delineate cardiac substructures automatically and study the relationship between substructure dose and treatment toxicities.	[32]
DL	217	NSCLC	∙DL segmentation model automatically segments cardiac substructures in cancer patients and can be used for cardiac radiation dose and radiation-induced mortality analysis.	[33]
DL	129	Breast cancer	∙DL provides an accurate and rapid segmentation of cardiac substructures in non-contrast CT images.	[34]
DL	127	NSCLC	∙DL models can outline the heart, ventricles, and great vessels in large datasets of chest CT images quickly, accurately, and consistently.	[35]
DL	49	Breast cancer	∙DL algorithms can obtain accurate estimates of radiation dose and dosimetry parameters for cardiac cavities, aortas, and coronary arteries automatically.	[36]
ANN	489	NSCLC	∙ANN was proposed to predict the incidence of CVDs after tumor resection and the ANN ensemble provided high performance.	[37]
RF	49,864	Prostate cancer	∙RF models outperformed multinomial logistic regression in predicting six-category COD including CVDs among prostate cancer patients.	[38]
RF	45,000	Breast cancer	∙RF had an accuracy of 70.23% in classifying five-category COD including CVDs in breast cancer patients.	[39]
RF	42,257	Lung cancer	∙RF models outperformed multinomial logistic regression in predicting five-category COD including CVDs among lung cancer patients.	[40]

ANN, artificial neural network; COD, causes of death; CVDs, cardiovascular diseases; DL, deep learning; ML, machine learning; LDCT, low-dose computed tomography; NSCLC, non-small cell lung cancer; RF, random forest; CT, computed tomography.

#### 2.1.1 Application of DL in Low-Dose Computed Tomography (LDCT)

To date, the application of DL in the field of cardio-oncology has focused mainly on LDCT [29, 30]. LDCT is effective in lung cancer screening in clinical trials [41, 42], and screening for CVD comorbidities in high-risk populations undergoing LDCT is vital for reducing overall mortality. In high-risk patients, LDCT based on DL can screen for lung cancer and estimate CVD risk simultaneously [29]. In addition, DL can be applied to conventional chest CT imaging to quantify left atrial volume and predict adverse outcomes: a study [30] found that DL-measured left atrial volume index was significantly related to the higher risks of new-onset atrial fibrillation (AF), heart failure, and major adverse cardiovascular events within five years. The predicted values showed high agreeability with manual quantification, reinforcing its potential clinical applicability. Furthermore, inputting the risk of plaque and coronary artery calcification (CAC) in chest LDCT images as scores into a hybrid neural network algorithm, which is a subtype of DL, can predict all-cause mortality in lung cancer patients. Compared with other neural networks that input images alone and conventional semi-automatic scoring methods, the hybrid neural network achieved better performance [31].

#### 2.1.2 Segmentation of Cardiac Substructures and Quantification of Radiation Therapy Dose

Radiation therapy for patients with thoracic malignant tumors can significantly reduce the local recurrence rate of patients. However, for patients whose tumors are in close proximity to the heart, the heart will be irradiated inevitably, potentially resulting in cardiac damage. Emerging evidence has shown that cardiotoxicity from radiotherapy may be associated with specific cardiac substructures, but manual delineation of these substructures can be challenging. Harms *et al*. [32] proposed a DL-based algorithm for automatic delineation of cardiac substructures, including ventricles, great vessels, coronary arteries, heart valves, and the whole heart, all of which could be segmented within five seconds. This provided a tool for investigating associations between the radiation dose on cardiac substructures and resultant toxicity. Additionally, Haq *et al*. [33] established and validated a similar model for twelve cardio-pulmonary substructures, reducing the segmentation time from one hour to ten seconds per patient; importantly, evaluation by radiation oncologists determined its clinical acceptability for use in treatment planning and clinical outcomes analysis. Recently, several studies have confirmed the reliability of DL models for automatic contouring of cardiovascular substructures on CT images of radiotherapy planning [34, 35, 36]. DL models can accurately and rapidly outline the heart and vessels in large datasets of chest CT images, and are expected to be time-saving in future clinical practice.

Analysis of confounding behavior of dosimetric variables predicts overall survival in cancer patients. Cardiac dosimetry of cardiac subvolumes is associated with decreased overall survival in patients with early-stage non-small cell carcinoma undergoing stereotactic ablative radiotherapy [43]. Using ML to optimize radiotherapy treatment plans for non-small cell cancer patients can reduce mean cardiac dose without increasing pulmonary dose [44]. It is thus feasible and desirable to apply these tools during radiotherapy to optimize dosimetric tradeoffs and minimize irradiation of the heart.

### 2.2 Artificial Neural Networks

ANNs are highly distributed and interconnected networks of computer elements modeled after biological nervous systems. ANNs simulate human neurons and combine neurons to form neural networks, use complex neural networks to make predictions, and perform regression analysis [45].

In patients with cancer, surgery can minimize tumor burden and is the most crucial treatment for solid tumors. However, postoperative inflammation may contribute to the development of adverse postoperative cardiovascular events, which are not rare and should be monitored closely. Studies have shown that tumor resection may be associated with cardiovascular complications such as arrhythmia, myocardial ischemia, and heart failure [46, 47, 48]. A limited number of neural networks trained for the same target can be assembled into a neural network ensemble, which can be used to predict morbidity. A recently proposed ANN ensemble predicted the occurrence of postoperative cardiovascular complications in non-small cell carcinoma patients undergoing pneumonectomy with satisfactory performance [37]. Before tumor surgery, attention should be paid to cardiovascular preparation and perioperative management. Such tools may aid pre-operative stratification of cardiovascular risk and optimization, which will likely reduce the risk of cardiovascular complications.

### 2.3 Random Forest

RF is a classifier that uses multiple trees to train and predict samples. The current application of RF in cardio-oncology mainly focuses on predicting the cause of cardiovascular death in cancer patients, and it has shown advantages in classification capabilities and accuracy compared with other ML and regression models [38]. Causes of death in cancer patients include cancer, non-cancer, infection, CVD, endocrine and blood diseases, and specific deaths because of other factors [38, 39, 40, 49]. A population-based study showed that CVD was the most common cause of death within the first year of cancer diagnosis [4]. It is thus essential to understand and prevent CVD in cancer patients. Three studies [38, 39, 40] used RF to predict causes of death, including CVD in breast cancer, lung cancer, and prostate cancer patients respectively, all of which found that RF was superior to multinomial logistic regression in predicting causes of death. RF allows systematic prediction of the cause of death in cancer patients and comprehensively analyzes the inter-relationships between risk factors, facilitating prevention of CVD amongst cancer patients.

## 3. ML in Cardiotoxicity

### 3.1 Ventricle—Cancer Treatment-Related Cardiac Dysfunction (CTRCD)

As the most common adverse drug events, drug-induced cardiovascular complications are the leading cause of discontinuation for many post-marketing drugs or of restrictions of their use [50]. A recent study [50] developed a combined classifier framework using several ML algorithms, including RF, support vector machine, k-nearest neighbors, and neural network. This classifier was validated for 63 anticancer agents using human pluripotent stem cell-derived cardiomyocytes, providing a powerful tool for systemic risk evaluation of drug-induced cardiovascular complications that can be applied to anticancer drug clinical trials and post-marketing surveillance [50].

CTRCD is defined as asymptomatic cardiac insufficiency or symptomatic heart failure manifested by adverse effects of cancer treatment on cardiac structure and function [51]. Cancer therapies, including conventional chemotherapeutics such as anthracyclines, as well as targeted therapies comprising small-molecule kinase inhibitors, agents targeting human epidermal growth factor receptor 2 (HER-2), and specific proteasome inhibitors, have been demonstrated to be associated with CTRCD [52, 53, 54, 55]. The prevention, detection and treatment of cardiac dysfunction patients before, during and after cancer therapy play significant roles in precision cardio-oncology.

Clinically, the diagnosis of CTRCD usually relies on standard echocardiographic monitoring, most often left ventricular ejection fraction (LVEF) and global longitudinal strain (GLS) [56]. However, inter-reader variability in reporting of LVEF with its implications in defining cancer therapy-related cardiotoxicity, and variability for GLS measurement among various ultrasound system vendors limit their application. Comfortingly, AI plays an important role in improving LVEF and GLS assessment and inter-vendor agreement. Studies have shown that automated magnetic resonance imaging (MRI)-based left ventricular contractility analysis tools can provide accurate estimates of cardiotoxic impairment associated with chemotherapy for cancer [57]. Troponin, natriuretic peptides, systolic global longitudinal and circumferential strain have been used to assess CTRCD risk. A recent prospective cohort study [58] identified an integrated approach combining three-dimensional echocardiographic LVEF, GLS, and global circumferential strain to diagnose CTRCD using a conditional inference tree model. Deep convolutional neural networks can provide important information on ventricular function, detection, and pathologies related to myocardial dysfunction, which can be used for routine monitoring to identify cardiotoxicity in cardiac image analysis [59, 60, 61, 62, 63, 64]. The DeepLabV3+ deep convolutional neural network and ResNet-50 backbone network can measure parameters such as left ventricular end-diastolic diameter and LVEF in cancer patients, suitable for automatic left ventricular quantification of cardiotoxicity [65]. A supervised ML model using RF regression can identify left ventricular segmental strain from acquired echocardiograms of patients undergoing cancer treatment and estimate the nadir of LVEF after treatment completion, thereby predicting CTRCD [66]. The cardiotoxicities identified by ML are summarized in Table 2 (Ref. [48, 58, 65, 66, 67, 68, 69, 70, 71, 72, 73, 74, 75, 76, 77, 78, 79, 80, 81, 82, 83]).

**Table 2.** **ML in cardiotoxicity**.

CVDs	Sample	Cancer types	ML types	Conclusions	Reference
CTRCD	136	Breast cancer	Conditional inference tree models	∙A combination of echocardiographic 3D left ventricular ejection fraction with 2D global circumferential strain and 2D global longitudinal strain can provide a quick diagnosis of CTRCD during routine surveillance.	[58]
CTRCD	42	Breast cancer	DeepLabV3 + deep CNN	∙Atrous deep CNN was validated for automated left ventricular chamber quantification and analysis of strain in cardiotoxicity detection.	[65]
CTRCD	237	Breast cancer	RF	∙Segmental strain measures identified by RF were applicable for CTRCD risk prediction in breast cancer patients receiving doxorubicin.	[66]
Arrhythmias	472	Colorectal cancer	Support vector machine	∙The support vector machine could model and count the major adverse cardiovascular events after a colorectal cancer operation.	[48]
Arrhythmias	1149	Chronic lymphocytic leukemia	AI-ECG algorithm using CNN	∙The AI-ECG algorithm using CNN could predict the occurrence of atrial fibrillation in chronic lymphocytic leukemia patients.	[67]
Arrhythmias	210,414	Prostate cancer	AI-ECG algorithm using CNN	∙Androgen deprivation therapy for prostate cancer was associated with changes in AI-ECG algorithm parameters.	[68]
Heart rate variability	77	Breast cancer, prostate cancer, colorectal cancer, lung cancer, and pancreatic cancer	RF, linear discriminant analysis, and naive bayes	∙ML algorithm heart rate variability parameters could be used as a reliable input to distinguish cancer patients from healthy controls.	[69]
ICIs related immunotoxicities	4960	Non-small cell lung cancer, melanoma, and renal cell carcinoma	Decision tree	∙ML could predict cardiac events in cancer patients receiving ICIs therapy.	[71]
ICIs related immunotoxicities	1152	Various cancers	Natural language processing	∙Natural language processing could identify ICIs treatment-related cardiotoxicity events.	[70]
Cardiomyopathy	1217	NA	AI-ECG	∙AI-ECG helps identify cancer survivors at increased risk of developing cardiomyopathy in the future.	[72]
Cardiomyopathy	471,9591	Lung cancer and breast cancer	ML algorithm	∙The ML analysis suggested that primary cancer types impacted the likelihood of stress cardiomyopathy, concomitant breast cancer and stress cardiomyopathy could significantly reduce mortality.	[73]
Coronary atherosclerosis	480	Lung cancer, prostate cancer, breast cancer, and hematological malignancies	Neural network ML	∙The innovative ML and statistical analysis suggested that specific cancer types could impact lesion severity in particular coronary vessels.	[74]
Coronary atherosclerosis	480	Various cancers	Neural network ML	∙Cancer patients had a lower burden of coronary atherosclerosis detected by coronary angiography.	[75]
CAC	15,915	Breast cancer	DL algorithm	∙Automated CAC scoring on DL was a powerful tool to identify breast cancer patients at increased risk of CVDs, especially coronary artery diseases.	[76]
CAC	12,332	Lung cancer	ML algorithm	∙Application of ML algorithm to LDCT scans to assess coronary calcium scores predicts CVD risk in cancer patients.	[77]
CAC	1825	NA	ML-based ECG	∙A ML-based ECG risk score can improve CVD risk stratification when added to CAC in cancer patients.	[78]
CAC	16,000	Breast cancer	DL algorithm	∙DL automatic algorithms have a good application in evaluating CAC scores and can be used to predict CVD morbidity and mortality in cancer patients.	[79]
CAC	428	Lung cancer	DL algorithm	∙Elevated CAC quantified by a DL model predicts mortality in cancer patients.	[80]
CAC	2289	Breast cancer	DL algorithm	∙DL was a reliable method to measure CAC in breast cancer patients undergoing CT scans.	[81]
CAC	1700	Breast cancer	DU-Net model	∙The DU-Net model was effective in breast arterial calcifications detection and could provide breast cancer patients with a low-cost risk assessment tool for CVDs.	[82]
CAC	840	Breast cancer	CNN	∙DL can effectively detect breast arterial calcification and assess cancer patients at high cardiovascular risks.	[83]

AI, artificial intelligence; CAC, coronary artery calcification; CNN, convolutional neural network; CTRCD, cancer treatment-related cardiac dysfunction; CVDs, cardiovascular diseases; DL, deep learning; ECG, electrocardiogram; ICIs, immune checkpoint inhibitors; ML, machine learning; RF, random forest; 2D, two dimensional; 3D, three dimensional; LDCT, low-dose computed tomography; CT, computed tomography. NA means that the article didn’t specify the cancer type of included patients.

### 3.2 Electrical System—Arrhythmias

Palpitation is a common complaint in cancer patients. Conventional cytotoxic chemotherapy, targeted drugs, and immunotherapies alike can lead to cancer therapy-related arrhythmias. The most common arrhythmia is AF, followed by ventricular arrhythmias, bradycardia and atrioventricular block [3, 54, 84, 85, 86, 87, 88].

The development of a rapid and inexpensive point-of-care method for AF screening using AI-enabled ECG has the potential to improve patient outcomes by enabling earlier detection and treatment of AF. Attia *et al*. [89] described the development of an AI-enabled ECG to identify patients with AF using standard 10-second, 12-lead ECGs. The AI algorithm was developed using a convolutional neural network and trained on a dataset of over 649,931 normal sinus rhythm ECGs from over 180,000 patients. The AI-enabled ECG was able to identify AF with an accuracy of 79.4% using a single ECG, and 83.3% when all ECGs acquired during the first month of each patient’s window of interest were included. Therefore, with high accuracy, AI-ECG could be used for AF screening in clinical practice. To evaluate the effectiveness of ML algorithms trained on ECG signals to predict patient outcomes after AF ablation, Tang *et al*. [90] used a convolutional neural network and a multimodal fusion framework to analyze data from 156 patients who underwent catheter ablation. The study highlights the importance of multimodal data analysis, which combines electrogram, ECG, and clinical features to improve the accuracy of prediction.

For cancer patients, AI-ECG is also a cost-effective and readily available tool for predicting cardiac arrhythmias. Christopoulos *et al*. [67] evaluated the role of AI-ECG in predicting AF in patients with ibrutinib-induced chronic lymphocytic leukemia and ibrutinib-independent AF. The AI-ECG algorithm, developed from a convolutional neural network, was used to monitor the rhythm of the two groups during the follow-up period, and the ECG characteristics of AF were used to predict the future risk of AF in the two groups of patients. The study demonstrated the feasibility and potential value of using AI-ECG in the management of patients with cancer. The role of AI algorithms in identifying androgen deprivation therapy (ADT)-induced changes in ECGs in prostate cancer patients has also been reported. A study [68] used a convolutional neural network to develop predictive signatures for cardiac pathologies, including the ability to predict the “estimated sex” of the patient. The results showed that patients who received ADT had a lower estimated male sex value in ECG compared to those who did not receive ADT, which was associated with decreased serum testosterone. This study highlights the potential of AI algorithms to detect changes in ECG parameters associated with ADT treatment for prostate cancer. The ability to monitor treatment effects and physiological changes non-invasively using ECGs has significant clinical implications, particularly for patients with cardiac morbidity and mortality associated with ADT. The findings also suggest the importance of considering the potential impact of ADT on serum testosterone levels and its association with ECG changes.

Drug-induced QT interval prolongation is a prevalent issue in various therapeutic interventions and can result in severe clinical consequences [91]. A relevant study [92] discussed the use of ML algorithms to predict the risk of drug-induced QT prolongation in inpatients. The researchers used harmonized data from the UCHealth electronic health record to compare multiple ML methods and found that a deep neural network demonstrated superior classification accuracy, which provided a reasonable predictive performance for identifying individuals with a high susceptibility to drug-induced QT prolongation. The further study compared the deep learning model, which had high accuracy but low interpretability, and the interpretable model based on cluster analysis, which was less accurate but more clinically applicable [93]. Both types of models have their own advantages and limitations, and that the choice of model should depend on the specific clinical context and the intended use of the prediction tool.

### 3.3 Autonomic Nervous System—Heart Rate Variability

It is common for cancer patients to exhibit autonomic dysfunction with reduced heart rate variability, which has prompted investigations into the potential value of heart rate variability (HRV) in cancer detection. Based on five-minute ECG recordings, Vigier *et al*. [69] showed that ML-analyzed algorithm heart rate variability parameters could be used to distinguish cancer patients from healthy individuals with an accuracy of 79%–85%. However, circadian rhythms may increase heart rate variability measurement variance due to differences in timing and equipment of the subjects’ ECG recordings, and sleep alterations may also cause changes in heart rate variability signatures. Uniform standardization of ECG recording time and equipment as well as questionnaires on sleep patterns are required. Future studies should be conducted in more diverse and more extensive samples of cancer patients to explore the robustness of heart rate variability-based cancer detection.

### 3.4 Myocardial Disorders

#### 3.4.1 Immune checkpoint inhibitors (ICIs)-Associated Myocarditis

ICIs have been one of the most promising types of anticancer drugs in recent years. ICIs therapy such as programmed death receptor-1(PD-1) or programmed death ligand-1(PD-L1) inhibitors can significantly improve the prognosis of cancer patients [94]. However, ICIs can induce a series of immune-related adverse events (irAEs) [95], including myocarditis, pericarditis, pericardial effusion, and acute vascular events [88, 96, 97, 98, 99], with myocarditis being the most frequently reported cardiac irAE [88, 100, 101, 102, 103]. Though uncommon, cardiac irAE may be fatal in up to 30% of cases [95], and the incidence of cardiac irAE associated with ICIs may be under-reported [96, 98, 104, 105, 106]. Therefore, it is important to identify risk factors for adverse cardiac events in patients receiving ICI therapy.

AI in the forms of ML and natural language processing (NLP) provides great analytical aptitude. The foundation of high-quality AI lies in data. As a considerable proportion of clinically relevant information is present in unstructured data, NLP assumes a critical role in extracting and analyzing information to inform decision-making, facilitate administrative reporting, and advance research efforts. This may be valuable when assessing complications or rare treatment-related events. Lu *et al*. [70] used NLP software to identify patients with various cancers treated with ICIs, and showed NLP could identify ICIs treatment-related cardiotoxicity events. NLP software can also be used to identify ICI-associated myocarditis in cancer patients and describe their clinical course and outcomes. Given the increasing popularity of ICIs treatment among various cancers, there is an unmet clinical need for identifying prognostic factors for myocarditis to facilitate clinical risk stratification, early diagnosis, and management.

Using a sizeable cross-sectional database of cancer patients, Heilbroner *et al*. [71] created a ML model for predicting cardiac events in PD-1/PD-L1-treated patients. The model comprehensively analyzed 356 potential risk factors and identified immunological, oncological, and cardiac risk factors associated with cardiac events. Although PD-1 treatment significantly increased the risk of cardiac events, PD-1 was not among the 40 most important predictors.

#### 3.4.2 Cardiomyopathy

Cardiomyopathy is a heterogeneous entity that includes numerous subtypes, such as dilated, hypertrophic, and restrictive cardiomyopathy [107]. Early identification of cancer patients at high risk of treatment-related cardiomyopathy may improve outcomes by intervening before heart failure development.

Güntürkün *et al*. [72] conducted a prospective study of 1217 childhood cancer survivors reaching adult age in the St Jude Lifetime Cohort, in which seven AI methods and an extreme gradient boosting approach were applied to 12-lead ECG to predict cardiomyopathy during a mean follow-up duration of 5.2 years. A resultant model based on ECG and clinical characteristics achieved a cross-validation area under curve (AUC) of 0.89, with a specificity of 81% and a sensitivity of 78%. AI-ECG may therefore help identify cancer survivors at increased risk of developing cardiomyopathy.

Stress cardiomyopathy is characterized by transient, reversible, local, or global myocardial dysfunction without ischemic perfusion defects [108, 109]. Catecholamine surge, epicardial coronary artery spasm and/or diffuse coronary vasoconstriction, and microvascular dysfunction are considered to be crucial mediating processes in the pathophysiology of stress cardiomyopathy [110]. Stress cardiomyopathy can be activated by various stressors, including infection, surgery, emotional or psychological stress, worsening chronic diseases, and medication [111]. Studies have shown that cancer patients with stress cardiomyopathy have a higher in-hospital mortality rate than those with only stress cardiomyopathy [112, 113]. However, a ML analysis by primary tumor types showed that stress cardiomyopathy was not associated with the in-hospital mortality of active cancer patients, with lung cancer and breast cancer both being associated with an increased likelihood of stress cardiomyopathy, and breast cancer patients with stress cardiomyopathy having a significantly reduced mortality [73]. Further prospective researches are needed to confirm these findings and reveal possible protective factors in breast cancer patients with stress cardiomyopathy.

### 3.5 Vessel Disorders

#### 3.5.1 Coronary Atherosclerosis

Cancer causes a hypercoagulable and pro-inflammatory state, sometimes accompanied by systemic infection which can lead to changes in inflammatory cytokines and massive release of chromatin. Meanwhile, cancer can increase the levels of peripheral blood neutrophils, predisposing to the formation of extracellular traps which are procoagulant and prothrombotic. The released chromatin and extracellular traps promote endothelial injury, followed by platelet aggregation, vasospasm, and possibly accelerated atherosclerosis [114, 115, 116, 117, 118]. ML can be used to assess whether specific malignancies can alter the natural progression and location of coronary artery diseases. A study [74] of both cancer patients and non-cancer patients undergoing coronary angiography showed that ML analysis was useful for identifying the locations of significant coronary stenoses. The study showed that lung cancer patients had higher odds of significant left anterior descending and right coronary artery stenoses than patients without lung cancer. However, the results remain controversial. Another ML-based analysis [75] showed that when compared to patients without cancer, patients with cancer had significantly fewer left anterior descending and left circumflex lesions, but were less likely to have multiple coronary arteries and acute left circumflex artery diseases. However, these findings were limited by potential bias by indication. More prospective studies should be conducted to reveal the longitudinal relationship between cancer and the development of coronary lesions.

#### 3.5.2 Coronary Artery Calcification

Measured from computed tomography scans, CAC is an independent risk factor for CVD [119, 120, 121]. Traditional CAC scoring is often manual, which is tedious and time-consuming [76]. Recently, several studies have shown that ML-based automated algorithms may be used for evaluating CAC scores, which, in turn, can be used to predict CVD morbidity and mortality in cancer patients [76, 77, 78, 79, 80, 81]. A DL-based CAC scoring algorithm was developed for automatic CAC scoring [122, 123], providing a rapid and low-cost tool for cancer patients at increased risks of CVD. Gernaat *et al*. [81] used a DL algorithm to score CAC automatically, and found that the prevalence of CAC in cancer patients was relatively high and increased with age. Comparisons with manual scoring confirmed the reliability of DL in measuring CAC. Similarly, Gal *et al*. [76] used a DL algorithm to automatically extract CAC scores, demonstrating a direct correlation between CAC scores and CVD risks among cancer patients.

Breast arterial calcification as detected by mammography may be evidence of general atherosclerosis, and may be a valuable marker of CVD [124]. Studies have shown that DL can be used to detect breast arterial calcification effectively and assess patients at high cardiovascular risks [82, 83], with performance similar or better than manual detection. Further large-scale studies are needed to test and improve these models across different experimental settings [125].

## 4. Prediction, Diagnosis, and Treatment of ML in Cardio-Oncology

### 4.1 Predicting the Risk of Cardiotoxicity

During chemotherapy and targeted therapy, cancer patients will develop adverse reactions of various tissues and organs in the body, among which cardiotoxicity is one of the most severe complications. Related studies have shown that ML can be used to predict the risk of cardiotoxicity in cancer patients after anticancer therapy. Li *et al*. [126] enrolled colorectal cancer patients treated with fluoropyrimidine and developed ML models including extreme gradient boosting, RF, and logistic regression to predict the subjects’ risk of cardiotoxicity. The study showed that among 36,030 colorectal cancer patients, 18.74% developed cardiotoxicity within 30 days after the first dose of fluoropyrimidine. All three ML models demonstrated high prediction accuracy with extreme gradient boosting having the best prediction performance. Thus, the ML model can accurately predict the occurrence of cardiotoxicity within a certain period after cancer patients start chemotherapy. Application of ML to positron emission tomography (PET) scans in cardio-oncology patients is also an emerging avenue. It has been demonstrated that risk prediction using ML applied to PET is more effective at predicting patients at high risk of major adverse cardiovascular events than logistic regression [127]. Additionally, PET scans in another study showed that coronary flow reserve was inversely related to radiation dose to specific coronary regions, which suggested that PET could identify coronary arteries damage after radiation therapy [128].

### 4.2 Screening Candidates for Specific Cancer Treatments

ML-based algorithms may be emerging diagnostic tools in cardio-oncology. For example, systolic dysfunction remains one of the significant side effects in patients with HER-2-positive breast cancer treated with trastuzumab. Therefore, current guidelines recommend that patients undergo echocardiography every three months during treatment. However, AI-augmented ECG using ML algorithms has good diagnostic performance in predicting abnormal ejection fraction while on trastuzumab therapy: a study [129] showed that the AI 12-lead ECG algorithm could reduce echocardiography by 15% when screening HER-2-positive breast cancer patients without missing a single patient with an LVEF of <40%, a degree of systolic dysfunction which usually warrants discontinuation of trastuzumab therapy.

Sometimes the lack of baseline LVEF can be a challenge when assessing the likelihood of CTRCD. ML algorithms can predict CTRCD in cancer patients based on clinically relevant variables, effectively circumventing the lack of LVEF. A classification model was trained to evaluate six cardiovascular outcomes, showing that clinical variables such as age, hypertension, blood glucose levels, creatinine and aspartate aminotransferase levels were all significantly associated with CTRCD [130]. Compared with traditional cardiac function monitoring methods, ML provides powerful tools for cardiac risk stratification in cancer patients by leveraging longitudinal, large-scale patient data from healthcare systems.

### 4.3 Potential Treatments of Cardiotoxicity Discovered by ML

Some cardio-protective agents can prevent or reduce cardiotoxicity during anticancer therapy. A recent meta-analysis [131] showed that angiotensin-converting enzyme inhibitors, angiotensin receptor blockers, and beta-blockers could preserve LVEF and protect against cardiotoxicity during trastuzumab and anthracycline treatment, with statistically significant outcomes with beta-blockers. Various anticancer drugs can produce off-targeted effects that negatively affect cardiac function and reduce LVEF. Heart failure with reduced ejection fraction should be concomitant with specific anticancer therapy to prevent cardiotoxicity. The American and European Cardio-Oncology Guidelines state that in patients with LVEF <40%, the use of HER-2 inhibitors is contraindicated except when there are no alternative cancer treatments available. For patients with LVEF <50% but ≥40%, the use of HER-2 inhibitors may be considered with a cardioprotective approach that includes angiotensin-converting enzyme inhibitors (or angiotensin receptor blockers) and/or beta-blockers [56]. In addition to the above three drugs, studies have shown that sacubitril/valsartan could also be used to protect against cardiotoxicity caused by anticancer drugs [132, 133].

An RF-based study [134] that performed exome sequencing of 289 childhood cancer survivors exposed to anthracyclines for at least three years, showed that almost 90% of patients without cardiotoxicity harbored rare/low-frequency variants in cardiac injury pathways that likely protected them from the damaging effects of anthracycline. In contrast, less than 50% of patients with cardiotoxicity harbored these variants. Compared with models using only clinical variables, cardiotoxicity risk prediction models incorporating clinical and genetic risk factors were more precise and had lower misclassification rates. The study using RF [134] found that *in vitro* gene inhibition of related pathways such as phosphoinositide-3-kinase regulatory subunit 2 (PI3KR2) and zinc finger protein 827 (ZNF827) protected cardiomyocytes from cardiotoxicity. The discovery of variant genes that protect against cardiotoxicity in cardiac injury pathways provides information for establishing predictive models for late-onset cardiotoxicity of anthracyclines, and autophagy gene targets to exploit cardio-protective drugs.

## 5. ML Revealed Inequalities and Disparities in Cardio-Oncology

### 5.1 Sex Disparities

Sex is an independent risk factor that increases the risk of adverse cardiovascular events in cancer patients. A recent systematic review and meta-analysis [135] found that among 13,975 patients with Hodgkin’s Lymphoma who received radiation therapy, cardiovascular events mortality was significantly higher in women than in men with an odds ratio of 3.74. Propensity scores use existing data to make statistically significant inferences and are of great value for real-world data analysis. However, the level of evidence in evidence-based medicine is still insufficient. As the accuracy and operational advantages of ML in large-scale medical data analysis are increasingly recognized, ML can be used to improve propensity scores, so that the two can be deeply combined to improve the selection accuracy of covariates in propensity scores [136].

One multicenter case-control study [137] used ML-augmented propensity score to analyze the outcomes of 30,195,722 hospitalized patients, revealing that percutaneous coronary intervention significantly reduced overall mortality, especially for cancer patients. Furthermore, percutaneous coronary intervention considerably reduced total hospitalization costs of cancer patients. However, a nationally representative case-control analysis using ML-generated propensity scores showed that women were less likely to undergo percutaneous coronary intervention and survive than men in cancer patients [138]. The above findings underscore the need for enhanced surveillance, and ML is expected to be better applied to monitor the incidence of adverse cardiovascular events in female cancer patients.

The inadequate representation of women in clinical trials, the higher dose of radiation required by female patients for specific types of cancer, and women’s higher degree of microvascular coronary artery diseases may account for the gender disparities [135, 139]. Further studies should focus on the age of the patients, treatment dose, duration, and methods of diagnosis for CVD to arrive at definitive conclusions about gender risks. The related guidelines also suggest optimizing gender equality in therapy access and outcomes. Future investigations on gender disparities should be expanded to other gender-discriminated groups, including gays, lesbians, bisexuals, asexuals, transgender, and intersex patients.

### 5.2 Racial Disparities

Though only early reports have been published, racial disparities may have adverse effects on the cardiovascular outcome of patients who receive cancer therapy. Limited data have shown that compared with non-Hispanic White, the incidence of cardiovascular adverse outcomes increases in black cancer patients. African American men patients are 2.5 times more likely to die from prostate cancer than white men [140], and black women patients with breast cancer had a 25% higher risk of cardiovascular death than non-Hispanic White [141]. A large case-control study [142] conducted a ML-optimized and propensity score-adjusted study about the mortality of cancer patients who received percutaneous coronary intervention treatment in the United States in 2016, revealing that although mortality was comparable, racial disparities in outcomes remained in cancer patients with percutaneous coronary intervention treatment. A case-control study [143] using propensity score analysis and neural network ML-augmented multivariable regression in cancer patients with CVDs showed a significant increase in mortality among Hispanics and Asians as compared with Caucasians. The current coronavirus disease-2019 pandemic has further enlarged the gap on the influence of the marginal population in history, strengthening the importance of determining and resolving this inequality [144].

The root causes of racial disparities are multifactorial, complex and interleaved. The previously described factors include racism, prejudice, limited health care, distrust of the medical profession due to historical experience, related genetic and molecular basis, and underrepresentation in clinical trials [145, 146, 147, 148]. One clinical trial study found that although African Americans constitute 22% of all US cancer patients, they only account for 3.1% of the trial participants [149].

Using ML approaches to identify racial disparities in patients with cardio-oncology diseases and improve the representation of different ethnic groups in clinical trials may therefore be critical for improving outcomes and reducing adverse CVD events. A multidisciplinary approach is needed to eliminate the racial disparities in cardio-oncology patients, including joint efforts of critical stakeholders, healthcare policymakers, clinicians, patients, and scientists [144].

## 6. Prospects and Challenges

### 6.1 The Trend of Revolution: Will ML Replace Clinicians?

At present, ML has a wide range of applications in the cardiovascular field, especially in many aspects of cardiac imaging, including but not limited to detection, characterization, and segmentation [150]. ML can take the place of many regular detection, characterization, and quantification work performed by clinicians using the cognitive ability and complete the integration of electronic medical records data mining [151, 152, 153]. It has been shown to outperform human experts in specific situations [31, 82]. This may raise a question amongst clinicians: is ML a threat or opportunity? Currently, most experts are of the opinion that although ML has dramatically improved its capabilities and applications, it will not replace clinicians at least in the near future [154, 155, 156]. Intelligent medical technology exists to improve patient management and support physician decision-making. ML has limitations, such as the need for large datasets for training and validation, and difficulty in identifying initial clustering patterns. As with any technology, ML is not infallible and there is a risk that it could fail. Some of the potential causes of ML failure include poor data quality, lack of domain knowledge, inappropriate modeling techniques, and overfitting of models to training data [157]. If we rely solely on advanced statistics, there is a risk of over-reliance on the models developed by ML algorithms, which can lead to poor decision-making. Human domain knowledge and expertise are also critical in interpreting the results of ML models and making informed decisions. In addition, the dehumanization of medicine is one of the most significant barriers to the application of intelligent medical technology [158]. Cancer not only brings physical pain to patients, but also many psychological and social challenges. Clinicians need to use appropriate information communication methods and content to provide psychological support and humanistic care. To this end, ML should assist clinicians as an adjunct, rather than being a replacement. Using AI is an iterative learning process, and the important thing is to harness and use this technique correctly and avoid misuse.

### 6.2 The Importance of Multidisciplinary Teams

Cardio-oncology is still in its infancy in many countries and its development has been lagging. The increasingly active research field of cardio-oncology warrants wider dissemination and integration into routine clinical practice. To this end, the formation of cardio-oncology teams with multidisciplinary input from cardiologists, oncologists, pharmacists, radiologists, psychologists, and nurse specialists is imperative. Besides, the opening of multidisciplinary joint diagnosis and treatment clinics in cardio-oncology is expected. Recently, the view of educating augmented doctors has been raised, and some universities have begun to innovate educational models to meet the need to train future doctors for the challenges of AI in medicine [158]. Although the risk of clinicians being replaced by ML in the near future is remote, clinicians are recommended to learn more about ML to rely on digital expertise and clinical experience to solve medical practice problems better. Researchers and clinicians can employ statistical software packages such as R or Python to construct and train ML models. The quantity of data necessary to build an ML model is contingent upon several factors, including the intricacy of the problem, the data quality, and the selected algorithm. Typically, larger datasets are preferred since they yield more informative content for the model to learn from and enhance the accuracy of predictions [159].

## 7. Conclusions

In conclusion, cardiotoxicities of cancer therapies cannot be ignored. ML plays a certain role in predicting, diagnosing and treating cardiotoxicities in cancer patients. As essential parts of ML, DL, RF, and ANN have tremendous applications in cardio-oncology. Inequalities exist in cardio-oncology, and ML is expected to optimize equality in treatment opportunities and outcomes. Medical education should cultivate interdisciplinary talents proficient in oncology, cardiovascular medicine and ML to meet future challenges.

## Abbreviations

ADT, androgen deprivation therapy; AF, atrial fibrillation; AI, artificial intelligence; ANN, artificial neural networks; CAC, coronary artery calcification; CTRCD, cancer treatment-related cardiac dysfunction; CVD, cardiovascular disease; DL, deep learning; ECG, electrocardiogram; GLS, global longitudinal strain; HER-2, human epidermal growth factor receptor 2; ICIs, immune checkpoint inhibitors; LDCT, low-dose computed tomography; LVEF, left ventricular ejection fraction; ML, machine learning; NLP, natural language processing; PD-1, programmed death receptor-1; PD-L1, programmed death ligand-1; PET, positron emission tomography; RF, random forest.

## References

[1] Lenneman CG, Sawyer DB (2016). Cardio-Oncology: An Update on Cardiotoxicity of Cancer-Related Treatment. *Circulation Research*.

[2] Yuan M, Zang L, Xu A, Gong M, Liu Q, Huo B (2021). Dynamic Changes of Serum Heart Type-Fatty Acid Binding Protein in Cancer Patients Treated With Immune Checkpoint Inhibitors. *Frontiers in Pharmacology*.

[3] Yuan M, Tse G, Zhang Z, Han X, Wu WKK, Li G (2018). The incidence of atrial fibrillation with trastuzumab treatment: A systematic review and meta-analysis. *Cardiovascular Therapeutics*.

[4] Sturgeon KM, Deng L, Bluethmann SM, Zhou S, Trifiletti DM, Jiang C (2019). A population-based study of cardiovascular disease mortality risk in US cancer patients. *European Heart Journal*.

[5] Vincent L, Leedy D, Masri SC, Cheng RK (2019). Cardiovascular Disease and Cancer: Is There Increasing Overlap. *Current Oncology Reports*.

[6] Koene RJ, Prizment AE, Blaes A, Konety SH (2016). Shared Risk Factors in Cardiovascular Disease and Cancer. *Circulation*.

[7] The Lancet Haematology (2022). Artificial intelligence-refinement and possibilities. *The Lancet Haematology*.

[8] Bachtiger P, Plymen CM, Pabari PA, Howard JP, Whinnett ZI, Opoku F (2020). Artificial Intelligence, Data Sensors and Interconnectivity: Future Opportunities for Heart Failure. *Cardiac Failure Review*.

[9] Chen D, Liu J, Zang L, Xiao T, Zhang X, Li Z (2022). Integrated Machine Learning and Bioinformatic Analyses Constructed a Novel Stemness-Related Classifier to Predict Prognosis and Immunotherapy Responses for Hepatocellular Carcinoma Patients. *International Journal of Biological Sciences*.

[10] Mintz Y, Brodie R (2019). Introduction to artificial intelligence in medicine. *Minimally Invasive Therapy & Allied Technologies: MITAT*.

[11] Nawaz MS, Shoaib B, Ashraf MA (2021). Intelligent Cardiovascular Disease Prediction Empowered with Gradient Descent Optimization. *Heliyon*.

[12] Lv H, Yang X, Wang B, Wang S, Du X, Tan Q (2021). Machine Learning-Driven Models to Predict Prognostic Outcomes in Patients Hospitalized With Heart Failure Using Electronic Health Records: Retrospective Study. *Journal of Medical Internet Research*.

[13] Kulkarni H, Thangam M, Amin AP (2021). Artificial neural network-based prediction of prolonged length of stay and need for post-acute care in acute coronary syndrome patients undergoing percutaneous coronary intervention. *European Journal of Clinical Investigation*.

[14] Visco V, Ferruzzi GJ, Nicastro F, Virtuoso N, Carrizzo A, Galasso G (2021). Artificial Intelligence as a Business Partner in Cardiovascular Precision Medicine: An Emerging Approach for Disease Detection and Treatment Optimization. *Current Medicinal Chemistry*.

[15] Tao K, Li J, Li J, Shan W, Yan H, Lu Y (2021). Estimation of Heart Rate Using Regression Models and Artificial Neural Network in Middle-Aged Adults. *Frontiers in Physiology*.

[16] Hadanny A, Shouval R, Wu J, Shlomo N, Unger R, Zahger D (2021). Predicting 30-day mortality after ST elevation myocardial infarction: Machine learning- based random forest and its external validation using two independent nationwide datasets. *Journal of Cardiology*.

[17] Velusamy D, Ramasamy K (2021). Ensemble of heterogeneous classifiers for diagnosis and prediction of coronary artery disease with reduced feature subset. *Computer Methods and Programs in Biomedicine*.

[18] Stevens LM, Linstead E, Hall JL, Kao DP (2021). Association Between Coffee Intake and Incident Heart Failure Risk: A Machine Learning Analysis of the FHS, the ARIC Study, and the CHS. *Circulation: Heart Failure*.

[19] Martin GP, Chew S, Palser TR (2017). The personal and the organisational perspective on iatrogenic harm: bridging the gap through reconciliation processes. *BMJ Quality & Safety*.

[20] Lyon AR, López-Fernández T, Couch LS, Asteggiano R, Aznar MC, Bergler-Klein J (2022). 2022 ESC Guidelines on cardio-oncology developed in collaboration with the European Hematology Association (EHA), the European Society for Therapeutic Radiology and Oncology (ESTRO) and the International Cardio-Oncology Society (IC-OS). *European Heart Journal*.

[21] Deo RC (2015). Machine Learning in Medicine. *Circulation*.

[22] Krittanawong C, Johnson KW, Rosenson RS, Wang Z, Aydar M, Baber U (2019). Deep learning for cardiovascular medicine: a practical primer. *European Heart Journal*.

[23] Al’Aref SJ, Anchouche K, Singh G, Slomka PJ, Kolli KK, Kumar A (2019). Clinical applications of machine learning in cardiovascular disease and its relevance to cardiac imaging. *European Heart Journal*.

[24] Bello GA, Dawes TJW, Duan J, Biffi C, de Marvao A, Howard LSGE (2019). Deep learning cardiac motion analysis for human survival prediction. *Nature Machine Intelligence*.

[25] Quer G, Arnaout R, Henne M, Arnaout R (2021). Machine Learning and the Future of Cardiovascular Care: JACC State-of-the-Art Review. *Journal of the American College of Cardiology*.

[26] Tison GH, Sanchez JM, Ballinger B, Singh A, Olgin JE, Pletcher MJ (2018). Passive Detection of Atrial Fibrillation Using a Commercially Available Smartwatch. *JAMA Cardiology*.

[27] Ardila D, Kiraly AP, Bharadwaj S, Choi B, Reicher JJ, Peng L (2019). End-to-end lung cancer screening with three-dimensional deep learning on low-dose chest computed tomography. *Nature Medicine*.

[28] Litjens G, Ciompi F, Wolterink JM, de Vos BD, Leiner T, Teuwen J (2019). State-of-the-Art Deep Learning in Cardiovascular Image Analysis. *JACC: Cardiovascular Imaging*.

[29] Aquino GJ, Chamberlin J, Mercer M, Kocher M, Kabakus I, Akkaya S (2022). Deep learning model to quantify left atrium volume on routine non-contrast chest CT and predict adverse outcomes. *Journal of Cardiovascular Computed Tomography*.

[30] Chao H, Shan H, Homayounieh F, Singh R, Khera RD, Guo H (2021). Deep learning predicts cardiovascular disease risks from lung cancer screening low dose computed tomography. *Nature Communications*.

[31] Yan P, Guo H, Wang G, Man RD, Kalra MK (2019). Hybrid Neural Networks for Mortality Prediction from LDCT Images. *Annual International Conference of the IEEE Engineering in Medicine and Biology Society. IEEE Engineering in Medicine and Biology Society. Annual International Conference*.

[32] Harms J, Lei Y, Tian S, McCall NS, Higgins KA, Bradley JD (2021). Automatic delineation of cardiac substructures using a region-based fully convolutional network. *Medical Physics*.

[33] Haq R, Hotca A, Apte A, Rimner A, Deasy JO, Thor M (2020). Cardio-pulmonary substructure segmentation of radiotherapy computed tomography images using convolutional neural networks for clinical outcomes analysis. *Physics and Imaging in Radiation Oncology*.

[34] Jin X, Thomas MA, Dise J, Kavanaugh J, Hilliard J, Zoberi I (2021). Robustness of deep learning segmentation of cardiac substructures in noncontrast computed tomography for breast cancer radiotherapy. *Medical Physics*.

[35] Garrett Fernandes M, Bussink J, Stam B, Wijsman R, Schinagl DAX, Monshouwer R (2021). Deep learning model for automatic contouring of cardiovascular substructures on radiotherapy planning CT images: Dosimetric validation and reader study based clinical acceptability testing. *Radiotherapy and Oncology: Journal of the European Society for Therapeutic Radiology and Oncology*.

[36] van Velzen SGM, Bruns S, Wolterink JM, Leiner T, Viergever MA, Verkooijen HM (2022). AI-Based Quantification of Planned Radiation Therapy Dose to Cardiac Structures and Coronary Arteries in Patients With Breast Cancer. *International Journal of Radiation Oncology, Biology, Physics*.

[37] Santos-García G, Varela G, Novoa N, Jiménez MF (2004). Prediction of postoperative morbidity after lung resection using an artificial neural network ensemble. *Artificial Intelligence in Medicine*.

[38] Wang J, Deng F, Zeng F, Shanahan AJ, Li WV, Zhang L (2020). Predicting long-term multicategory cause of death in patients with prostate cancer: random forest versus multinomial model. *American Journal of Cancer Research*.

[39] Deng F, Huang J, Yuan X, Cheng C, Zhang L (2021). Performance and efficiency of machine learning algorithms for analyzing rectangular biomedical data. *Laboratory Investigation; a Journal of Technical Methods and Pathology*.

[40] Deng F, Zhou H, Lin Y, Heim JA, Shen L, Li Y (2021). Predict multicategory causes of death in lung cancer patients using clinicopathologic factors. *Computers in Biology and Medicine*.

[41] Aberle DR, Adams AM, Berg CD, Black WC, Clapp JD, National Lung Screening Trial Research Team (2011). Reduced lung-cancer mortality with low-dose computed tomographic screening. *The New England Journal of Medicine*.

[42] Bach PB, Mirkin JN, Oliver TK, Azzoli CG, Berry DA, Brawley OW (2012). Benefits and harms of CT screening for lung cancer: a systematic review. *JAMA*.

[43] Chan ST, Ruan D, Shaverdian N, Raghavan G, Cao M, Lee P (2020). Effect of Radiation Doses to the Heart on Survival for Stereotactic Ablative Radiotherapy for Early-stage Non-Small-cell Lung Cancer: An Artificial Neural Network Approach. *Clinical Lung Cancer*.

[44] Bitterman DS, Selesnick P, Bredfeldt J, Williams CL, Guthier C, Huynh E (2022). Dosimetric Planning Tradeoffs to Reduce Heart Dose Using Machine Learning-Guided Decision Support Software in Patients with Lung Cancer. *International Journal of Radiation Oncology, Biology, Physics*.

[45] Grande-Fidalgo A, Calpe J, Redón M, Millán-Navarro C, Soria-Olivas E (2021). Lead Reconstruction Using Artificial Neural Networks for Ambulatory ECG Acquisition. *Sensors (Basel, Switzerland)*.

[46] Haapio E, Kiviniemi T, Irjala H, Koivunen P, Airaksinen JKE, Kinnunen I (2016). Incidence and predictors of 30-day cardiovascular complications in patients undergoing head and neck cancer surgery. *European Archives of Oto-rhino-laryngology*.

[47] Vogelsang RP, Søby JH, Tolstrup MB, Burcharth J, Ekeløf S, Gögenur I (2021). Associations between malignancy and cardiovascular complications following emergency laparotomy - A retrospective cohort study. *Surgical Oncology*.

[48] Tian Q, Zheng L, Wang CR, Xie W, Yang X (2020). Modeling and analysis of the effect of wenxin granule on relieving arrhythmia after operation in patients with colorectal cancer. *Basic and Clinical Pharmacology and Toxicology*.

[49] Kaalby L, Al-Najami I, Deding U, Berg-Beckhoff G, Steele RJC, Kobaek-Larsen M (2022). Cause of Death, Mortality and Occult Blood in Colorectal Cancer Screening. *Cancers*.

[50] Cai C, Fang J, Guo P, Wang Q, Hong H, Moslehi J (2018). In Silico Pharmacoepidemiologic Evaluation of Drug-Induced Cardiovascular Complications Using Combined Classifiers. *Journal of Chemical Information and Modeling*.

[51] Herrmann J, Lenihan D, Armenian S, Barac A, Blaes A, Cardinale D (2022). Defining cardiovascular toxicities of cancer therapies: an International Cardio-Oncology Society (IC-OS) consensus statement. *European Heart Journal*.

[52] Agunbiade TA, Zaghlol RY, Barac A (2019). Heart Failure in Relation to Tumor-Targeted Therapies and Immunotherapies. *Methodist DeBakey Cardiovascular Journal*.

[53] Agunbiade TA, Zaghlol RY, Barac A (2019). Heart Failure in Relation to Anthracyclines and Other Chemotherapies. *Methodist DeBakey Cardiovascular Journal*.

[54] Herrmann J (2020). Adverse cardiac effects of cancer therapies: cardiotoxicity and arrhythmia. *Nature Reviews. Cardiology*.

[55] Kenigsberg B, Jain V, Barac A (2017). Cardio-oncology Related to Heart Failure: Epidermal Growth Factor Receptor Target-Based Therapy. *Heart Failure Clinics*.

[56] Alexandre J, Cautela J, Ederhy S, Damaj GL, Salem JE, Barlesi F (2020). Cardiovascular Toxicity Related to Cancer Treatment: A Pragmatic Approach to the American and European Cardio-Oncology Guidelines. *Journal of the American Heart Association*.

[57] Kar J, Cohen MV, McQuiston SA, Figarola MS, Malozzi CM (2020). Fully automated and comprehensive MRI-based left-ventricular contractility analysis in post-chemotherapy breast cancer patients. *The British Journal of Radiology*.

[58] Esmaeilzadeh M, Urzua Fresno CM, Somerset E, Shalmon T, Amir E, Fan CPS (2022). A Combined Echocardiography Approach for the Diagnosis of Cancer Therapy-Related Cardiac Dysfunction in Women With Early-Stage Breast Cancer. *JAMA Cardiology*.

[59] Bai W, Sinclair M, Tarroni G, Oktay O, Rajchl M, Vaillant G (2018). Automated cardiovascular magnetic resonance image analysis with fully convolutional networks. *Journal of Cardiovascular Magnetic Resonance*.

[60] Duchateau N, King AP, De Craene M (2020). Machine Learning Approaches for Myocardial Motion and Deformation Analysis. *Frontiers in Cardiovascular Medicine*.

[61] Fahmy AS, El-Rewaidy H, Nezafat M, Nakamori S, Nezafat R (2019). Automated analysis of cardiovascular magnetic resonance myocardial native T_1_ mapping images using fully convolutional neural networks. *Journal of Cardiovascular Magnetic Resonance: Official Journal of the Society for Cardiovascular Magnetic Resonance*.

[62] Zhang N, Yang G, Gao Z, Xu C, Zhang Y, Shi R (2019). Deep Learning for Diagnosis of Chronic Myocardial Infarction on Nonenhanced Cardiac Cine MRI. *Radiology*.

[63] Zhang J, Gajjala S, Agrawal P, Tison GH, Hallock LA, Beussink-Nelson L (2018). Fully Automated Echocardiogram Interpretation in Clinical Practice. *Circulation*.

[64] Kar BJ, Cohen MV, McQuiston SP, Malozzi CM (2021). A deep-learning semantic segmentation approach to fully automated MRI-based left-ventricular deformation analysis in cardiotoxicity. *Magnetic Resonance Imaging*.

[65] Karr J, Cohen M, McQuiston SA, Poorsala T, Malozzi C (2021). Validation of a deep-learning semantic segmentation approach to fully automate MRI-based left-ventricular deformation analysis in cardiotoxicity. *The British Journal of Radiology*.

[66] Demissei BG, Fan Y, Qian Y, Cheng HG, Smith AM, Shimamoto K (2021). Left ventricular segmental strain and the prediction of cancer therapy-related cardiac dysfunction. *European Heart Journal. Cardiovascular Imaging*.

[67] Christopoulos G, Attia ZI, Noseworthy PA, Call TG, Ding W, Leis JF (2020). Use of Artificial Intelligence Electrocardiography to Predict Atrial Fibrillation (AF) in Patients with Chronic Lymphocytic Leukemia (CLL). *Blood*.

[68] Breen W, Carter R, Johnson P, Routman DM, Noseworthy P, Herrmann J (2020). An artificial intelligence-enabled analysis of ECG changes after androgen deprivation therapy (ADT) for prostate cancer. *Journal of Clinical Oncology*.

[69] Vigier M, Vigier B, Andritsch E, Schwerdtfeger AR (2021). Cancer classification using machine learning and HRV analysis: preliminary evidence from a pilot study. *Scientific Reports*.

[70] Lu DJ, Kamrava M, McArthur HL, Reckamp K, Tamarappoo B, Atkins KM (2020). Using Natural Language Processing to Identify Patients with Immune Checkpoint Inhibitor-Associated Myocarditis. *International Journal of Radiation Oncology Biology Physics*.

[71] Heilbroner SP, Few R, Mueller J, Chalwa J, Charest F, Suryadevara S (2021). Predicting cardiac adverse events in patients receiving immune checkpoint inhibitors: a machine learning approach. *Journal for Immunotherapy of Cancer*.

[72] Güntürkün F, Akbilgic O, Davis RL, Armstrong GT, Howell RM, Jefferies JL (2021). Artificial Intelligence-Assisted Prediction of Late-Onset Cardiomyopathy Among Childhood Cancer Survivors. *JCO Clinical Cancer Informatics*.

[73] Javaid AI, Monlezun DJ, Iliescu G, Tran P, Filipescu A, Palaskas N (2021). Stress cardiomyopathy in hospitalized patients with cancer: machine learning analysis by primary malignancy type. *ESC Heart Failure*.

[74] Boone DL, Monlezun DJ, Cervoni-Curet F, Chohan J, Sunny J, Bhatti K (2018). Malignancy type impacts coronary lesion severity and location: Multivariable regression with concurrent machine learning-backed case-control analysis. *Circulation*.

[75] Balanescu DV, Monlezun DJ, Donisan T, Boone D, Cervoni-Curet F, Palaskas N (2019). A Cancer Paradox: Machine-Learning Backed Propensity-Score Analysis of Coronary Angiography Findings in Cardio-Oncology. *The Journal of Invasive Cardiology*.

[76] Gal R, van Velzen SGM, Hooning MJ, Emaus MJ, van der Leij F, Gregorowitsch ML (2021). Identification of Risk of Cardiovascular Disease by Automatic Quantification of Coronary Artery Calcifications on Radiotherapy Planning CT Scans in Patients With Breast Cancer. *JAMA Oncology*.

[77] Stemmer A, Shadmi R, Bregman-Amitai O, Chettrit D, Blagev D, Orlovsky M (2020). Using machine learning algorithms to review computed tomography scans and assess risk for cardiovascular disease: Retrospective analysis from the National Lung Screening Trial (NLST). *PLoS ONE*.

[78] Kumar SS, Al-Kindi S, Tashtish N, Rajagopalan V, Fu P, Rajagopalan S (2021). Machine learning applied on ECG improves cardiovascular risk prediction when added to coronary artery calcium scoring in patients with cancer. *Circulation*.

[79] Emaus MJ, Išgum I, van Velzen SGM, van den Bongard HJGD, Gernaat SAM, Lessmann N (2019). Bragatston study protocol: a multicentre cohort study on automated quantification of cardiovascular calcifications on radiotherapy planning CT scans for cardiovascular risk prediction in patients with breast cancer. *BMJ Open*.

[80] Atkins KM, Weiss J, Zeleznik R, Bitterman DS, Chaunzwa TL, Huynh E (2022). Elevated Coronary Artery Calcium Quantified by a Validated Deep Learning Model From Lung Cancer Radiotherapy Planning Scans Predicts Mortality. *JCO Clinical Cancer Informatics*.

[81] Gernaat SAM, van Velzen SGM, Koh V, Emaus MJ, Išgum I, Lessmann N (2018). Automatic quantification of calcifications in the coronary arteries and thoracic aorta on radiotherapy planning CT scans of Western and Asian breast cancer patients. *Radiotherapy and Oncology: Journal of the European Society for Therapeutic Radiology and Oncology*.

[82] AlGhamdi M, Abdel-Mottaleb M, Collado-Mesa F (2020). DU-Net: Convolutional Network for the Detection of Arterial Calcifications in Mammograms. *IEEE Transactions on Medical Imaging*.

[83] Wang J, Ding H, Bidgoli FA, Zhou B, Iribarren C, Molloi S (2017). Detecting Cardiovascular Disease from Mammograms With Deep Learning. *IEEE Transactions on Medical Imaging*.

[84] Alvi RM, Frigault MJ, Fradley MG, Jain MD, Mahmood SS, Awadalla M (2019). Cardiovascular Events Among Adults Treated With Chimeric Antigen Receptor T-Cells (CAR-T). *Journal of the American College of Cardiology*.

[85] Chandrasekhar S, Fradley MG (2019). QT Interval Prolongation Associated With Cytotoxic and Targeted Cancer Therapeutics. *Current Treatment Options in Oncology*.

[86] Escudier M, Cautela J, Malissen N, Ancedy Y, Orabona M, Pinto J (2017). Clinical Features, Management, and Outcomes of Immune Checkpoint Inhibitor-Related Cardiotoxicity. *Circulation*.

[87] Fradley MG, Gliksman M, Emole J, Viganego F, Rhea I, Welter-Frost A (2019). Rates and Risk of Atrial Arrhythmias in Patients Treated With Ibrutinib Compared With Cytotoxic Chemotherapy. *The American Journal of Cardiology*.

[88] Mahmood SS, Fradley MG, Cohen JV, Nohria A, Reynolds KL, Heinzerling LM (2018). Myocarditis in Patients Treated With Immune Checkpoint Inhibitors. *Journal of the American College of Cardiology*.

[89] Attia ZI, Noseworthy PA, Lopez-Jimenez F, Asirvatham SJ, Deshmukh AJ, Gersh BJ (2019). An artificial intelligence-enabled ECG algorithm for the identification of patients with atrial fibrillation during sinus rhythm: a retrospective analysis of outcome prediction. *The Lancet*.

[90] Tang S, Razeghi O, Kapoor R, Alhusseini MI, Fazal M, Rogers AJ (2022). Machine Learning-Enabled Multimodal Fusion of Intra-Atrial and Body Surface Signals in Prediction of Atrial Fibrillation Ablation Outcomes. *Circulation: Arrhythmia and Electrophysiology*.

[91] Roden DM (2004). Drug-induced prolongation of the QT interval. *The New England Journal of Medicine*.

[92] Simon ST, Mandair D, Tiwari P, Rosenberg MA (2021). Prediction of Drug-Induced Long QT Syndrome Using Machine Learning Applied to Harmonized Electronic Health Record Data. *Journal of Cardiovascular Pharmacology and Therapeutics*.

[93] Simon ST, Trinkley KE, Malone DC, Rosenberg MA (2022). Interpretable Machine Learning Prediction of Drug-Induced QT Prolongation: Electronic Health Record Analysis. *Journal of Medical Internet Research*.

[94] Reck M, Rodríguez-Abreu D, Robinson AG, Hui R, Csőszi T, Fülöp A (2016). Pembrolizumab versus Chemotherapy for PD-L1-Positive Non-Small-Cell Lung Cancer. *The New England Journal of Medicine*.

[95] Brumbaugh AD, Narurkar R, Parikh K, Fanucchi M, Frishman WH (2019). Cardiac Immune-Related Adverse Events in Immune Checkpoint Inhibition Therapy. *Cardiology in Review*.

[96] Behling J, Kaes J, Münzel T, Grabbe S, Loquai C (2017). New-onset third-degree atrioventricular block because of autoimmune-induced myositis under treatment with anti-programmed cell death-1 (nivolumab) for metastatic melanoma. *Melanoma Research*.

[97] Drobni ZD, Alvi RM, Taron J, Zafar A, Murphy SP, Rambarat PK (2020). Association Between Immune Checkpoint Inhibitors With Cardiovascular Events and Atherosclerotic Plaque. *Circulation*.

[98] Norwood TG, Westbrook BC, Johnson DB, Litovsky SH, Terry NL, McKee SB (2017). Smoldering myocarditis following immune checkpoint blockade. *Journal for Immunotherapy of Cancer*.

[99] Varricchi G, Galdiero MR, Marone G, Criscuolo G, Triassi M, Bonaduce D (2017). Cardiotoxicity of immune checkpoint inhibitors. *ESMO Open*.

[100] Bersanelli M, Giannarelli D, Castrignanò P, Fornarini G, Panni S, Mazzoni F (2018). INfluenza Vaccine Indication During therapy with Immune checkpoint inhibitors: a transversal challenge. The INVIDIa study. *Immunotherapy*.

[101] Moslehi JJ (2016). Cardiovascular Toxic Effects of Targeted Cancer Therapies. *The New England Journal of Medicine*.

[102] Thavendiranathan P, Zhang L, Zafar A, Drobni ZD, Mahmood SS, Cabral M (2021). Myocardial T1 and T2 Mapping by Magnetic Resonance in Patients With Immune Checkpoint Inhibitor-Associated Myocarditis. *Journal of the American College of Cardiology*.

[103] Zhang L, Awadalla M, Mahmood SS, Nohria A, Hassan MZO, Thuny F (2020). Cardiovascular magnetic resonance in immune checkpoint inhibitor-associated myocarditis. *European Heart Journal*.

[104] Gujral DM, Mouhayar EN, Bhattacharyya S (2020). Cardiac Adverse Events Related to Immune Checkpoint Inhibitors. *JACC: Case Reports*.

[105] Tay RY, Blackley E, McLean C, Moore M, Bergin P, Gill S (2017). Successful use of equine anti-thymocyte globulin (ATGAM) for fulminant myocarditis secondary to nivolumab therapy. *British Journal of Cancer*.

[106] Zhang L, Reynolds KL, Lyon AR, Palaskas N, Neilan TG (2021). The Evolving Immunotherapy Landscape and the Epidemiology, Diagnosis, and Management of Cardiotoxicity: *JACC: CardioOncology* Primer. *JACC: CardioOncology*.

[107] Brieler J, Breeden MA, Tucker J (2017). Cardiomyopathy: An Overview. *American Family Physician*.

[108] Eres R, Bolton I, Lim M, Lambert G, Lambert E (2021). Cardiovascular responses to social stress elicited by the cyberball task. *Heart and Mind*.

[109] Ho JSY, Ng TYM, Cen S, Sia CH, Chan PF, Yeo TC (2021). Metastatic ovarian cancer presenting as takotsubo cardiomyopathy: A case report. *Heart and Mind*.

[110] Wittstein IS, Thiemann DR, Lima JAC, Baughman KL, Schulman SP, Gerstenblith G (2005). Neurohumoral features of myocardial stunning due to sudden emotional stress. *The New England Journal of Medicine*.

[111] Sharkey SW, Windenburg DC, Lesser JR, Maron MS, Hauser RG, Lesser JN (2010). Natural history and expansive clinical profile of stress (tako-tsubo) cardiomyopathy. *Journal of the American College of Cardiology*.

[112] Cammann VL, Sarcon A, Ding KJ, Seifert B, Kato K, Di Vece D (2019). Clinical Features and Outcomes of Patients With Malignancy and Takotsubo Syndrome: Observations From the International Takotsubo Registry. *Journal of the American Heart Association*.

[113] Joy PS, Guddati AK, Shapira I (2018). Outcomes of Takotsubo cardiomyopathy in hospitalized cancer patients. *Journal of Cancer Research and Clinical Oncology*.

[114] Blann AD, Dunmore S (2011). Arterial and venous thrombosis in cancer patients. *Cardiology Research and Practice*.

[115] Chen KC, Liao YC, Wang JY, Lin YC, Chen CH, Juo SHH (2015). Oxidized low-density lipoprotein is a common risk factor for cardiovascular diseases and gastroenterological cancers via epigenomical regulation of microRNA-210. *Oncotarget*.

[116] Demers M, Krause DS, Schatzberg D, Martinod K, Voorhees JR, Fuchs TA (2012). Cancers predispose neutrophils to release extracellular DNA traps that contribute to cancer-associated thrombosis. *Proceedings of the National Academy of Sciences of the United States of America*.

[117] Giza DE, Marmagkiolis K, Mouhayar E, Durand JB, Iliescu C (2017). Management of CAD in Patients with Active Cancer: the Interventional Cardiologists’ Perspective. *Current Cardiology Reports*.

[118] Kotla S, Zhang A, Imanishi M, Ko KA, Lin SH, Gi YJ (2021). Nucleus-mitochondria positive feedback loop formed by ERK5 S496 phosphorylation-mediated poly (ADP-ribose) polymerase activation provokes persistent pro-inflammatory senescent phenotype and accelerates coronary atherosclerosis after chemo-radiation. *Redox Biology*.

[119] Lehker A, Mukherjee D (2021). Coronary Calcium Risk Score and Cardiovascular Risk. *Current Vascular Pharmacology*.

[120] Matos D, Ferreira AM, de Araújo Gonçalves P, Gama F, Freitas P, Guerreiro S (2021). Coronary artery calcium scoring and cardiovascular risk reclassification in patients undergoing coronary computed tomography angiography. *Revista Portuguesa De Cardiologia*.

[121] Xia C, Vonder M, Sidorenkov G, Den Dekker M, Oudkerk M, van Bolhuis JN (2021). Cardiovascular Risk Factors and Coronary Calcification in a Middle-aged Dutch Population: The ImaLife Study. *Journal of Thoracic Imaging*.

[122] Lessmann N, van Ginneken B, Zreik M, de Jong PA, de Vos BD, Viergever MA (2018). Automatic Calcium Scoring in Low-Dose Chest CT Using Deep Neural Networks With Dilated Convolutions. *IEEE Transactions on Medical Imaging*.

[123] van Velzen SGM, Lessmann N, Velthuis BK, Bank IEM, van den Bongard DHJG, Leiner T (2020). Deep Learning for Automatic Calcium Scoring in CT: Validation Using Multiple Cardiac CT and Chest CT Protocols. *Radiology*.

[124] Bui QM, Daniels LB (2019). A Review of the Role of Breast Arterial Calcification for Cardiovascular Risk Stratification in Women. *Circulation*.

[125] Trimboli RM, Codari M, Guazzi M, Sardanelli F (2019). Screening mammography beyond breast cancer: breast arterial calcifications as a sex-specific biomarker of cardiovascular risk. *European Journal of Radiology*.

[126] Li C, Chen L, Chou C, Ngorsuraches S, Qian J (2022). Using Machine Learning Approaches to Predict Short-Term Risk of Cardiotoxicity Among Patients with Colorectal Cancer After Starting Fluoropyrimidine-Based Chemotherapy. *Cardiovascular Toxicology*.

[127] Juarez-Orozco LE, Knol RJJ, Sanchez-Catasus CA, Martinez-Manzanera O, van der Zant FM, Knuuti J (2020). Machine learning in the integration of simple variables for identifying patients with myocardial ischemia. *Journal of Nuclear Cardiology: Official Publication of the American Society of Nuclear Cardiology*.

[128] Dreyfuss AD, Bravo PE, Koumenis C, Ky B (2019). Precision Cardio-Oncology. *Journal of Nuclear Medicine: Official Publication, Society of Nuclear Medicine*.

[129] Attia ZI, Kapa S, Noseworthy PA, Asirvatham SJ, Pellikka PA, Ladewig DJ (2019). Monitoring of Breast Cancer Patients Left Ventricular Ejection Fraction Using Ai Augmented Electrocardiogram. *Heart Rhythm*.

[130] Zhou Y, Hou Y, Hussain M, Brown SA, Budd T, Tang WHW (2020). Machine Learning-Based Risk Assessment for Cancer Therapy-Related Cardiac Dysfunction in 4300 Longitudinal Oncology Patients. *Journal of the American Heart Association*.

[131] Lewinter C, Nielsen TH, Edfors LR, Linde C, Bland JM, LeWinter M (2022). A systematic review and meta-analysis of beta-blockers and renin-angiotensin system inhibitors for preventing left ventricular dysfunction due to anthracyclines or trastuzumab in patients with breast cancer. *European Heart Journal*.

[132] Wang Y, Tse G, Roever L, Liu T (2020). Sacubitril/valsartan in the treatment of cancer therapy-related cardiac dysfunction. *International Journal of Cardiology*.

[133] Duraes AR, de Souza Lima Bitar Y, Neto MG, Mesquita ET, Chan JS, Tse G (2022). Effectiveness of sacubitril-valsartan in patients with cancer therapy-related cardiac dysfunction: a systematic review of clinical and preclinical studies. *Minerva Medica*.

[134] Chaix MA, Parmar N, Kinnear C, Lafreniere-Roula M, Akinrinade O, Yao R (2020). Machine Learning Identifies Clinical and Genetic Factors Associated With Anthracycline Cardiotoxicity in Pediatric Cancer Survivors. *JACC: CardioOncology*.

[135] Khalid Y, Fradley M, Dasu N, Dasu K, Shah A, Levine A (2020). Gender disparity in cardiovascular mortality following radiation therapy for Hodgkin’s lymphoma: a systematic review. *Cardio-oncology*.

[136] Thomason N, Monlezun DJ, Javaid A, Filipescu A, Koutroumpakis E, Shobayo F (2022). Percutaneous Coronary Intervention in Patients With Gynecological Cancer: Machine Learning-Augmented Propensity Score Mortality and Cost Analysis for 383,760 Patients. *Frontiers in Cardiovascular Medicine*.

[137] Monlezun DJ, Lawless S, Palaskas N, Peerbhai S, Charitakis K, Marmagkiolis K (2021). Machine Learning-Augmented Propensity Score Analysis of Percutaneous Coronary Intervention in Over 30 Million Cancer and Non-cancer Patients. *Frontiers in Cardiovascular Medicine*.

[138] Kim JW, Monlezun DJ, Palaskas N, Cilingiroglu M, Marmagkiolis K, Iliescu CA (2021). Sex disparities in cardio-oncology treatment and mortality: Propensity score nationally representative case-control analysis with machine learning augmentation of over 30 million hospitalizations. *Catheterization and Cardiovascular Interventions*.

[139] Ohman RE, Yang EH, Abel ML (2021). Inequity in Cardio-Oncology: Identifying Disparities in Cardiotoxicity and Links to Cardiac and Cancer Outcomes. *Journal of the American Heart Association*.

[140] Jemal A, Siegel R, Ward E, Murray T, Xu J, Smigal C (2006). Cancer statistics, 2006. *CA: a Cancer Journal for Clinicians*.

[141] Sengupta R, Honey K (2020). *AACR Cancer Disparities Progress Report 2020*: Achieving the Bold Vision of Health Equity for Racial and Ethnic Minorities and Other Underserved Populations. *Cancer Epidemiology, Biomarkers & Prevention: a Publication of the American Association for Cancer Research, Cosponsored by the American Society of Preventive Oncology*.

[142] Kim JW, Monlezun DJ, Palaskas N, Cilingiroglu M, Marmagkiolis K, Iliescu CA (2021). Racial and income disparities persist in pci among cardio-oncology patients despite comparable mortality: Propensity score nationally representative case-control analysis with machine learning augmentation of 30 million+ hospitalizations. *Catheterization and Cardiovascular Interventions*.

[143] Agrawal N, Palaskas NL, Cilingiroglu M, Marmagkiolis K, Dhoble A, Arain SA (2021). Racial and income inequities in cardiovascular disease in cancer versus non-cancer patients: propensity score and machine learning augmented nationally representative case-control study of mortality and cost among 30 million hospitalizations dominique monlezun. *Circulation*.

[144] Fazal M, Malisa J, Rhee JW, Witteles RM, Rodriguez F (2021). Racial and Ethnic Disparities in Cardio-Oncology: A Call to Action. *JACC: CardioOncology*.

[145] Challa AA, Calaway AC, Cullen J, Garcia J, Desai N, Weintraub NL (2021). Cardiovascular Toxicities of Androgen Deprivation Therapy. *Current Treatment Options in Oncology*.

[146] Hashimoto Y, Shiina M, Dasgupta P, Kulkarni P, Kato T, Wong RK (2019). Upregulation of miR-130b Contributes to Risk of Poor Prognosis and Racial Disparity in African-American Prostate Cancer. *Cancer Prevention Research (Philadelphia, Pa.)*.

[147] Heaphy CM, Joshu CE, Barber JR, Davis C, Zarinshenas R, De Marzo AM (2020). Racial Difference in Prostate Cancer Cell Telomere Lengths in Men with Higher Grade Prostate Cancer: A Clue to the Racial Disparity in Prostate Cancer Outcomes. *Cancer Epidemiology, Biomarkers & Prevention: a Publication of the American Association for Cancer Research, Cosponsored by the American Society of Preventive Oncology*.

[148] Karakas C, Wang C, Deng F, Huang H, Wang D, Lee P (2017). Molecular mechanisms involving prostate cancer racial disparity. *American Journal of Clinical and Experimental Urology*.

[149] Loree JM, Anand S, Dasari A, Unger JM, Gothwal A, Ellis LM (2019). Disparity of Race Reporting and Representation in Clinical Trials Leading to Cancer Drug Approvals From 2008 to 2018. *JAMA Oncology*.

[150] Chan J, Auffermann WF (2020). Artificial Intelligence in Cardiopulmonary Imaging. *Advances in Clinical Radiology*.

[151] Kohli M, Prevedello LM, Filice RW, Geis JR (2017). Implementing Machine Learning in Radiology Practice and Research. *American Journal of Roentgenology*.

[152] Krittanawong C (2018). The rise of artificial intelligence and the uncertain future for physicians. *European Journal of Internal Medicine*.

[153] Lakhani P, Prater AB, Hutson RK, Andriole KP, Dreyer KJ, Morey J (2018). Machine Learning in Radiology: Applications Beyond Image Interpretation. *Journal of the American College of Radiology*.

[154] Pesapane F, Codari M, Sardanelli F (2018). Artificial intelligence in medical imaging: threat or opportunity? Radiologists again at the forefront of innovation in medicine. *European Radiology Experimental*.

[155] Topol EJ (2019). High-performance medicine: the convergence of human and artificial intelligence. *Nature Medicine*.

[156] Verghese A, Shah NH, Harrington RA (2018). What This Computer Needs Is a Physician: Humanism and Artificial Intelligence. *JAMA*.

[157] Krittanawong C, Zhang H, Wang Z, Aydar M, Kitai T (2017). Artificial Intelligence in Precision Cardiovascular Medicine. *Journal of the American College of Cardiology*.

[158] Briganti G, Le Moine O (2020). Artificial Intelligence in Medicine: Today and Tomorrow. *Frontiers in Medicine*.

[159] Jordan MI, Mitchell TM (2015). Machine learning: Trends, perspectives, and prospects. *Science (New York, N.Y.)*.

